# Lessons Learnt from the EU Response to NCDs: A Content Analysis on Building Resilient Post-COVID Health Systems

**DOI:** 10.3390/healthcare9121659

**Published:** 2021-11-30

**Authors:** Rana Orhan, Martina Paric, Katarzyna Czabanowska

**Affiliations:** 1 Department of International Health, Care and Public Health Research Institute CAPHRI, Faculty of Health, Medicine and Life Sciences, Maastricht University, 6229 GT Maastricht, The Netherlands; m.paric@maastrichtuniversity.nl (M.P.); kasia.czabanowska@maastrichtuniversity.nl (K.C.); 2 The Association of Schools of Public Health in the European Region, 1150 Brussels, Belgium; 3 Department of Health Policy Management, Institute of Public Health, Faculty of Health Sciences, Jagiellionian University, 31-066 Krakow, Poland

**Keywords:** non-communicable diseases, COVID-19 pandemic, resilience, public health systems, European Health Union, European public health

## Abstract

Non-communicable diseases (NCDs) impose a heavy burden on the healthcare system of countries in the European Union (EU). An estimated 91.3% of all deaths and 86.6% of DALYs in the EU-28 were attributable to NCDs. It is imperative that the EU act on mitigating this challenging health issue and help create trajectories for building resilient health systems. Using qualitative analysis, this study examines the question of how the European Commission 2019–2024 is planning to mitigate the impact of NCDs on health systems, while taking into account the COVID-19 pandemic. A content analysis of 44 documents communicating the European Commission’s position on the issue was done. In vivo coding was performed using the software package ATLAS.ti 9. Unique codes were simplified and grouped into main themes. Five main themes were identified: ‘health plan’, ‘COVID-19’, ‘future direction’, ‘collaboration and solidarity’, and ‘persuasion’. This study shows that the European Commission is emphasising the impact of the pandemic and the relevance of policies tackling NCDs. By calling for more cross- and multi-sectoral collaboration, the Commission hopes to create the right climate for a European framework for cooperation, which can help develop EU-wide resilient health systems.

## 1. Introduction

Non-communicable diseases (NCDs) impose a heavy burden on national healthcare systems in the European Union (EU). In 2019, an estimated 91.08% (4.9 million) of all deaths and 87.55% of all Disability-Adjusted Life Years (DALYs) in the EU were attributable to NCDs [1]. At least 80% of all heart disease, diabetes, and stroke cases, and 40% of cancer cases could be prevented by tackling major risk factors, such as tobacco use, alcohol use, unhealthy diet, physical inactivity, and environmental factors [2]. It is imperative that the EU act on mitigating this challenging health issue and help create trajectories for building resilient health systems.

At the same time, NCDs are a great burden on the economy of EU Member States. The economic cost of NCDs can be divided into three categories: (1) costs at the level of individuals and households (e.g., increased disabilities, decreased household income, and increased expenditures), (2) healthcare delivery costs (e.g., more need for use of healthcare services and high medical treatment costs), and (3) costs to national economies (e.g., absenteeism in labour and lower tax revenues) [3]. The four major NCDs (cardiovascular disease, cancer, chronic respiratory disease, and diabetes mellitus) account for at least 25% of national health spending and an economic loss of 2% of the gross domestic product in EU Member States [4]. It is expected that the economic cost of NCDs will steeply increase in the future, whereby mortality rates will fall but years lived with a chronic illness will rise.

Due to long-term underinvestment by Member States, national healthcare systems have been unable to protect population health by implementing programs focused on prevention, early diagnosis, screening, treatment, and rehabilitation for NCDs [5]. This was highlighted by the COVID-19 pandemic, which has put enormous pressure on healthcare systems in several ways. People living with NCDs are more vulnerable to becoming ill or dying from COVID-19 infection. At the same time, healthcare services for the prevention and treatment of NCDs have been disrupted, which is expected to result in excess deaths [5].

The EU has a long track record of policies aimed at tackling NCDs. Among other actions, the European Commission supports Member States in reaching the nine voluntary targets of the Global Monitoring Framework for NCDs. These targets were formulated by the World Health Organisation (WHO) and aim to reduce premature death from the four major NCDs by 25% in 2025, compared to 2010 [6,7]. In 2018, the Commission established the Steering Group on Health Promotion, Disease Prevention and Management of Non-Communicable Diseases to support Member States in reaching Sustainable Development Goal 3 (particularly, reducing premature non-communicable disease mortality) and promoting cooperation between the Member States to address challenges caused by NCDs [8]. More recently, in the first quarter of 2021, the EU has launched several initiatives linked with NCDs: the EU4Health programme, Europe’s Beating Cancer Plan (EBCP), and Horizon Europe including its Cancer Mission. The EU4Health programme is the EU’s response to COVID-19, supporting health promotion and disease prevention “by addressing health risk factors and health determinants” [9]. Europe’s Beating Cancer Plan aims to tackle the entire disease pathway of cancer, including cancer prevention, early detection, diagnosis and treatment, and improving the quality of life of cancer patients and survivors [10]. The Cancer Mission of Horizon Europe sets out 13 recommendations to achieve “more than 3 million lives saved, living longer and better” by 2030 [11].

As shown by a geopolitical analysis of 194 countries, among former Soviet states and OECD members, the EU27 saw the highest level of implementation of non-communicable disease policies from 2015 to 2020 [12]. It is evident that the EU has been preparing itself for reducing the detrimental impact of public health issues on European society. Yet, at the same time as NCD-related initiatives were launched, many more were set up to tackle the COVID-19 pandemic. The WHO Regional Office for Europe developed a list of NCD-specific responses during the COVID-19 pandemic to help address the needs of those living with NCDs or at risk of NCDs [13]. It is unknown, however, whether the European Commission’s focus on tackling NCDs was kept up in the course of the pandemic.

There have been several qualitative studies using content or discourse analysis in relation to policy documents; however, most have focused on identifying policy problems and solutions rather than explaining policymaking dynamics [14,15,16]. Additionally, the usefulness of this approach for policy analysis has been highlighted in several publications [17,18]. To our knowledge, no studies have focused on understanding EU approaches concerning NCD health policies. Using qualitative analysis, this research examines the question of how the European Commission 2019–2024 is planning to mitigate the impact of NCDs on health systems, while taking into account the COVID-19 pandemic. The results of this study can prove valuable for European policymakers by providing them with the knowledge on how to develop more resilient health systems through a more effective response to NCDs in times of similar crises.

## 2. Materials and Methods

The research question was approached using content analysis, which is a qualitative research technique uniquely suited for analysing textual data in the contexts of their use. Moreover, it is a suitable approach when dealing with large amounts of text that needs to be categorised into manageable content categories with similar meanings [19,20]. This methodology can be applied to understand how NCDs are framed and how an EU narrative is constructed by the European Commission.

### 2.1. Sources of Data

A search strategy to identify relevant literature, understand the studied context, and help establish coding categories while performing the content analysis was applied for data collection [19]. The search for textual data was conducted throughout February 2021 and included all documents that were published since the start of the Von der Leyen Commission on the 1 December 2019. All documents that communicated the European Commission’s position on the issue of tackling NCDs, such as policy documents, the European Commission work programme, speeches, event outcomes, answers by the Commissioners to parliamentary questions, parliamentary committee meetings, minutes, policy briefs, press releases, and other documents relating to relevant meetings, were considered. The search was conducted on the Commission’s website and the website of the European Parliament. Documents relating to parliamentary committee meetings and answers by the Commission to parliamentary questions ended up being excluded as a unit for analysis, as these documents often referred to already published material and policies. Similarly, event outcomes and minutes were excluded as they did not give substantial information to help answer the research question. Bias in data collection was eliminated as various types of documents were consulted for data collection to ensure sampling validity [19].

The following key terms and their combinations were used for the search: EU4Health, Horizon Europe’s Cancer Mission, Europe’s Beating Cancer Plan, European Health Union, NCDs, cancer, tobacco, alcohol, nutrition, lung health, and heart disease. These key terms were chosen because they directly pertained to the Commission’s strategy on NCDs, referred to determinants of NCDs, or directly described associated diseases. Documents not directly relating to NCDs were excluded. Namely, those focusing on asbestos, antibiotics, pharmaceutical strategy, substances (e.g., sodium, radon, phone radiation, pesticides, microplastics), air pollution, antimicrobial resistance, vaccines, COVID-19 (unless related to cancer or NCDs), Horizon Europe in general, health research in general, and health inequalities (including access to healthcare) in general. This choice was made in order to gather the most relevant material for analysis, with strong links to NCDs.

After applying the inclusion and exclusion criteria, a total of 79 documents were identified for analysis. These consisted of policy documents (*n* = 14), press releases (*n* = 13), speeches (*n* = 33), and statements (*n* = 19). A further 35 documents were excluded during the coding process as full-text analysis showed that they did not adhere to the inclusion criteria, resulting in a total of 44 documents for final analysis (Appendix A, Table A1).

### 2.2. Data Analysis

All documents were imported into the qualitative data analysis research software ATLAS.ti 9. R.O. and M.P. independently pilot coded six documents and met in order to refine and agree on preliminary codes. Afterwards, these same documents were sent to KC with the preliminary codes. This was done in order to ensure intercoder reliability, which allowed for the creation of consensus on coding instructions [21]. R.O. and M.P. proceeded to code the remaining texts, with scheduled regular meetings to check each other’s work and resolve any uncertainties. Coding was done sentence by sentence, often relying on in vivo codes, which were later grouped into content categories. These content categories, referred to as ‘codes’, consisted of in vivo codes that were abstracted and grouped together. Codes were then clustered into descriptive themes with a high level of abstraction. Content categories and their interpretation were discussed by R.O., M.P., and K.C. in order to ensure consensus and saturation for major themes.

## 3. Results

Grouping all of the codes resulted in five themes: ‘health plan’, ‘COVID-19’, ‘future direction’, ‘collaboration and solidarity’, and ‘persuasion’ (Table 1). The prominent theme in the analysed documents was ‘Health plan’ (codes’ frequency = 1031). The most frequent codes were ‘Elements of Europe’s Beating Cancer Plan’ (belonging to the ‘Health plan’ theme), ‘Implementing a modern approach to cancer’ (idem), and ‘Working together on all levels’ (belonging to the ‘Collaboration and solidarity’ theme). The section below will go over the different themes, including some excerpts from the analysed documents (Appendix A).

### 3.1. Health Plan

*“Understanding the complexity of cancer and the role of factors and determinants (e.g., lifestyle, environment, workplace exposure, but also sex/gender and age) is important for developing effective preventive measures. Some factors are known to play a role in the development of cancers but their precise impact is not clear, whereas other causal factors remain to be discovered. Moreover, changing human behaviour has proven to be a challenge. Therefore, more understanding is needed of how people perceive health threats and cancer risks, how they behave accordingly and how unhealthy behaviours can be sustainably changed.”*

(Document No. 39)

This theme grouped together all in vivo codes referring to the Commission’s description of Europe’s Beating Cancer Plan (EBCP), affiliated health initiatives, desired results concerning this NCD strategy, areas and motivations for action, as well as any utterances concerning financing or funding. By far the most prominent topic was the EBCP itself and analysis showed details on the aim, ambitions, and specific strategies of the Plan. Hence, all references to the Plan were grouped together in a single code. The interlinkage with other policies, such as Horizon Europe’s Cancer Mission, the Farm to Fork Strategy, and the EU Action Plan on Childhood Obesity, was constantly made, linking the Plan to the research, climate, and other health-related agendas of the European Commission. For instance, the Commission also has the ambition for a Tobacco Free Generation, and this code was also included as another element of Europe’s Beating Cancer Plan.

Secondly, a separate code was formed concerning the Commission’s aims to tackle cancer by implementing a modern approach. This means cross-cutting, cross- and multi-sectoral action to improve the quality of health systems. They state that rapid innovations in cancer care are needed and acknowledge the potential of new technologies, such as telemedicine, big data analysis, and personalised medicine. Effective cancer prevention strategies, such as healthy diets and physical activity, are also mentioned. Additionally, focusing on a more patient-centred approach, health education, the development of the health workforce, and European guidelines would form elements of implementing a modern approach to cancer. Furthermore, this code signifies how the Commission frames their approach to NCDs as “new” and “modern” because they acknowledge how complex these diseases are.

Thirdly, the Commission mentions that there are wide disparities in healthcare and existing inequalities across Member States. Enabling equal access to healthcare was seen as key in the health plan. The EU’s role was hereby described as facilitating data availability in Member States and advising on the latest developments.

This leads to the fourth code in this theme, namely how cancer is framed as a public health priority since the beginning of the current Commission’s mandate, and continues to be during the COVID-19 pandemic. This prioritisation was reconfirmed by the launch of Europe’s Beating Cancer Plan on 3 February 2021. Moreover, public health as a field itself was stated to be a priority for the European Union. This was mentioned specifically in the context of Europe’s recovery from the COVID-19 pandemic.

*“The public needs a clear message that public health is a priority area for Europe’s recovery; that targeted investment in crisis preparedness and stronger health systems is essential for the future; that we are mobilising all possible resources to strengthen our defences—including, crucially, funding, which in many cases is a critical determinant of feasibility.”*

(Document No. 13)

Finally, in terms of monetary means to tackle NCDs, the European Commission mentions the allocation of funding to implement policies, such as Europe’s Beating Cancer Plan, the EU4Health programme, and Next Generation EU. The Commission also responded to calls from civil society to invest more in tackling NCDs, such as with World Mental Health Day. By mentioning how these different initiatives are financed, where the funding is coming from, or by calling for further investment, the Commission further anchors the Plan as a reality.

### 3.2. COVID-19

*“The Mission on Cancer will be a major driving force to apply the lessons from the current COVID-19 crisis to find solutions to the cancer challenge and beyond. We have seen an unprecedented willingness in technology adaptation, collaboration across sectors and borders, including extensive data sharing, genuine communication and alignment between healthcare and research, remarkably shortening the implementation of research findings and the ability to mobilise and allocate considerable funding resources at short notice.”*

(Document No. 39)

The second theme centred around all utterances about COVID-19, its impact, lessons learnt, and specifically how the pandemic showed why prioritising public health and collectively planning actions concerning crisis preparedness is necessary. Additionally, in this theme, codes referring to the role of the EU and EU institutions were emphasised as a separate category because such utterances were directly tied to how the pandemic impacted the operations of the EU.

The first code in this theme reframed the impact of the pandemic to emphasise the relevance of policies tackling NCDs and developing an EU-wide resilient health system. The European Commission formulated the EU4Health programme to respond to the crisis and prepare for the future. The pandemic showed that there is a lack of readiness and preparedness at all levels, but also showed the importance of health and that quick progress is possible. Horizon Europe’s Cancer Mission is stated to be a major driving force to apply the lessons from the pandemic to tackle cancer.

The second code is slightly different from the previous one, as it is more descriptive and not a call-to-action. Rather, the Commission highlights the impact of the crisis on the EU and worldwide, as well as how the pandemic brought to light specific challenges. Overcoming these challenges posed by the pandemic is mentioned, but the focus is more on the weaknesses in our health systems and gaps in surveillance systems.

*“We are also making sure that our two EU Agencies who have been at the forefront of our work, the European Centre for Disease Prevention and Control (ECDC) and the European Medicines Agency (EMA), have the necessary capacity to fully play the role we need them to. This will be a step change for EU’s collective capacity to respond and coordinate.”*

(Document No. 26)

To bolster health capacities, the European Commission developed the EU Vaccines Strategy. This third code was deemed significant as the Commission’s utterances on the pandemic significantly shifted towards creating a common strategy with a focus on vaccines. Although this strategy is only applicable to COVID-19 vaccines, the shortage of other vaccinations also received attention during the pandemic, indicating that the Commission sees an expanding public health role for the EU and EU institutions.

This brings us to the final code in this theme, concerning this new role of the EU, ECDC, and EMA. The European Centre for Disease Prevention and Control (ECDC) has been acknowledged for their fundamental role in the COVID-19 pandemic, by establishing risk assessments in the early stages. To prepare better for future pandemics or health emergencies, the European Commission has been discussing extending the mandate of the ECDC, along with the European Medicines Agency (EMA). Similar to the previous code, this one was also deemed significant as it shows how the Commission frames who will be taking action specifically. The Commission also proposed setting up the EU Health Emergency Response Authority (HERA), which aims to support in times of crises, such as the COVID-19 pandemic.

### 3.3. Future Direction

*“A stronger European Health Union would allow EU countries to work together more efficiently to detect, prepare and respond collectively to future health threats. … the Health Union will go beyond crisis management, making health systems as a whole more resilient.”*

(Document No. 14)

This theme grouped codes on what it is the Commission wants to accomplish. The first code collected all utterances that indicated the intent to build a European Health Union, extending the ECDC and EMA’s mandate are part of building a “bold, strong, future European Health Union”. The Commission stated they need the commitment of Member States for this to move towards a common approach, with EU-wide health policies, more robust systems, and a focus on the collective power of the EU. The importance of coordinated EU action in order to build such a Union was mentioned often.

The second code in this theme refers to taking a holistic approach to public health issues, by including a wide range of policy areas. This code often came back when describing the different elements of the health plan, specifically when these elements called for action in different policy areas simultaneously. In a few documents, this was described as a necessary “paradigm shift”.

*“The third essential element is how we deal with health from now on. Do we need to shift the paradigm in terms of health policy in the European Union?”*

(Document No. 24)

Thirdly, building a strong resilient health system was mentioned throughout most (policy) documents. According to the Commission, the COVID-19 pandemic has shown how fragile our health systems are. Therefore, they aim to make the health systems more resilient and to improve the crisis management capacity with policies, such as the EU4Health programme. However, also not-COVID-19-related policies are aimed at increasing resilience. The word ‘resilience’ was often mentioned by the Commission, specifically when imagining or describing what it is the Commission wants to achieve for Europe.

Finally, a separate code was created for cross-border cooperation. To build resilience against future crises, cross-border cooperation needs to be increased. The Commission often refers to the potential of cross-border healthcare and strengthening European measures across borders in order to tackle NCDs. For instance, when it comes to potentially regulating cross-border alcohol and tobacco purchases. Furthermore, the pandemic highlighted a need for better cooperation across borders.

### 3.4. Collaboration and Solidarity

*“The coronavirus outbreak taught us that we need to make some structural improvements in how we work together.”*

(Document No. 10)

During the analysis, it became clear that the Commission emphasises collaboration and solidarity as an important strategy for realising their plans. The first code in this theme refers to any utterances on “working together”. The need for support from Member States and other stakeholders and joining forces were stated multiple times by the Commission. This was emphasised often through a variety of phrases, which indicated that only collectively can the Commission’s Plan be realised. Building strong partnerships and a European framework for cooperation are seen as steps towards the coalition ‘we’ need.

Secondly, the Commission often described the plan as an ambitious undertaking. Due to this description, they further emphasise the need for collaboration. In several documents, there was mentioning of the “ambitious” Horizon Europe’s Cancer Mission, the “ambitious” Europe’s Beating Cancer Plan, the “ambitious” EU4Health programme, and the “ambitious” new EU Vaccines Strategy. By using this phrasing, the Commission frames its health plan to tackle NCDs as unprecedented and unique.

The third code in this theme was created because the European Commission repeatedly mentioned European solidarity. Solidarity is said to save lives. By solidarity, they refer to solidarity between Member States and between the EU and third countries. Solidarity, along with equity, is stated to be a European value. Similar to the code resilience, solidarity is another specific concept that the Commission places central in its approach to framing the issue.

*“One of the core values across the European Union is the shared commitment to universal access to high-quality care financed on the basis of equity and solidarity.”*

(Document No. 39)

Finally, to place urgency on collaborating and exercising solidarity, the European Commission stated in several documents the need to be more proactive, to collectively do more, and to strive for more. This code often occurs in conjunction with describing the plan as ambitious, as it emphasises how achievable the initiative is. This code is different from the previous codes within this theme because it emphasises the capacity for further action.

### 3.5. Persuasion

*“… I tackled breast cancer both as a patient and as an advocate. … and they shared stories that I personally know so well.”*

(Document No. 1)

*“Everyone has a friend, a colleague or a relative who’s gone through this. Everyone has experienced the same sense of sadness and helplessness.”*

(Document No. 4)

The last theme identified techniques of persuasion adopted by the Commission in order to convince the audience of the necessity of their plan to tackle NCDs and build a resilient health system.

Personalisation was used often to persuade the audience in speeches or other documents. The documents that were analysed showed the Commission used specific language to play on the audience’s imagination to recall a situation where they encountered a loved one’s suffering. In such cases, personalisation was used to convince the audience of the necessity of this plan.

Furthermore, to persuade the audience, data is provided in all policy documents and in nearly all speeches, press releases, and news items. The code ‘provided data’ included any kind of data, such as statistics on the prevalence of NCDs. This data aimed to contribute to the understanding of the scope of the problem.

Thirdly, the Commission emphasises that there is a need for a shared long-term strategic vision that is not only European but global. The Commission states that they have the political will to tackle NCDs and that this will was not impacted due to the pandemic. References to a shared vision and commitment were placed in a separate code because this specific phrasing was often used by the Commission to create a sense of conviction.

*“So we need a shared vision for the future of health in Europe. … For us this vision is a compass which will guide us on the next steps. We are now getting to work to turn this vision into tangible reality.”*

(Document No. 19)

Additionally, a code was created to group together utterances relating to the Commission’s insistence to act now, to not waste time, and to wake up. They emphasize the need for urgency when tackling NCDs, claiming that we are moving in the right direction but need to do more. This sort of phrasing was used often and shows a clear tendency from the Commission to build on positive momentum whenever possible.

*“But we can and must do better in other areas—and COVID-19 has shown us that we have no time to waste.”*

(Document No. 19)

Finally, the last code grouped together all utterances concerning the burden of NCDs to persuade the audience. The impact of cancer on the EU, the rise of NCDs, the pressures on limited resources, and the framing of NCDs as a huge threat are mentioned. This code is separate from provided data because it is descriptive rather than specific. Both were often used simultaneously in order to convince the audience.

## 4. Discussion

This study identified five main themes including ‘health plan’, ‘COVID-19’, ‘future direction’, ‘collaboration and solidarity’, and ‘persuasion’ by using a qualitative approach aimed at understanding how the Commission is planning to mitigate the impact of NCDs on health systems. It shows that the Commission is emphasising the impact of the pandemic and the relevance of policies tackling NCDs for developing EU-wide resilient health systems.

The clearest action towards this goal is the creation of a “bold, strong, future European Health Union”, a first step being the mandate extension of the existing health authorities ECDC and EMA, and the launch of a new health authority, HERA. In the analysed documents, the Commission stated what a stronger European Health Union would bring (i.e., more resilient health systems), and that it would mean more collective power for the EU and the need for Member States’ commitment. A European Health Union would not only strengthen public health in Europe but would also have an impact on global public health as the EU’s competitive position is bolstered. Additionally, current EU health law stipulates that the EU is obliged to protect public health in all EU policies and regulations, including external policies, which have a further positive impact on global public health [22].

However, what a European Health Union would or should entail is not made clear in any of the analysed documents. To “work together more efficiently to detect, prepare and respond collectively to future health threats” indeed goes beyond crisis management, but what else would the mandate for the EU entail?

As De Ruijter points out, does a European Health Union mean that citizens from all Member States would have access to high-quality healthcare [23]? Our analysis shows that the Commission acknowledges the existence of wide disparities in healthcare across the Member States. Enabling equal access to healthcare is seen as key in tackling cancer and other NCDs. However, other than facilitating data and advising on the latest developments, no role has been proposed for the Commission. Therefore, our results align with De Ruijter that access to the same high-quality healthcare throughout Europe seems unlikely, or at least, that there is no concrete idea communicated by the Commission. The Commission is calling for solidarity between Member States by stating that there is a shared commitment to access to high-quality healthcare. This implies sustaining the status quo of health system competencies, i.e., with Member States. Furthermore, it is the Member States’ responsibility to eliminate inequalities within their own national health systems in order to meet the expectations of the Commission concerning ‘high-quality care’ in the EU.

This brings us to the question of whether the European Health Union will continue to be an ambition for a future far beyond the current Commission’s mandate: is the extension of the ECDC and EMA mandates, and the creation of HERA, in terms of legislation sufficient to increase European health systems’ resilience?

The EU4Health programme was launched as a financial response to the COVID-19 pandemic, in order to prepare the EU for future crises, including future actions concerning NCDs. The Commission also responded positively to calls from civil society organisations to invest more in tackling NCDs. However, reality shows that financial support for (public) health civil society is being limited, creating a paradox at a time of public health crisis [24]. As The Lancet describes, “COVID-19 must stimulate far greater political action to overcome inertia around NCDs” [25]. We could argue that a European Health Union in which the competencies of the EU and Member States change is needed to be able to tackle NCDs effectively. Nonetheless, the EU’s role could also be to “provide what it already does”, as Clemens and Brand argue [26]. In this case, institutional innovation within the current competencies of the EU could already prove to be sufficient.

Our findings show that a lot of emphasis is placed on EU-wide health policies, more robust health systems, and the collective power of the EU. However, there are no concrete actions coupled with these concepts, aside from implying it would be a natural evolution of events as more cross- and multi-sectoral collaboration occurs. It is implied that there is a disconnect between Member States’ needs and the Commission’s Plan, since there are no actionable points for Member States in terms of addressing national health policy at the administrative level, what kind of leadership is needed, where change should be stimulated, or what specific measurable results should be observed. For these reasons, there is a danger that the Plan might just remain a financial aid for local projects with little dissemination, a high administrative burden, and with little cross-over towards building a European Health Union.

Our analysis also showed a strong interlinkage made with other policy areas, such as climate change and environmental policy, employment and workforce development, trade, and other legal policies, to name a few. A holistic approach to public health issues that is cross-cutting, cross- and multi-sectoral is seen as a necessary paradigm shift to improve the quality of health systems, which resonates with Allen et al. [12]. For example, the ongoing pandemic has disproportionately affected people living with NCDs, many of them tobacco induced. The strong link between tobacco use and higher risks of severe complications and death from COVID-19 was answered by the ambition for a Tobacco Free Generation (as mentioned in Europe’s Beating Cancer Plan) and by referring to the potential of cross-border measures, such as better regulation of tobacco and alcohol purchases [27].

Furthermore, we gained insight into the persuasion techniques of the Commission. They used personalisation, data, a shared vision, the creation of urgency, and emphasised the burden of NCDs as ways to create a window of opportunity to take action. As shown in their talk of a shared mission, the Commission stated that the political will to tackle NCDs was not impacted due to the COVID-19 pandemic. They also referred to their health plan as unprecedented and unique. We question whether this really is the case, or whether it is merely a financial response, whereby Member States can apply for funding for innovation in health, which is similar to trends in the past. The Commission’s need to do more proactively and collectively, and to strive for more, brings the worry that there is a mismatch between the ambitions at EU level and the realities at Member State level. While Europe’s Beating Cancer Plan states cancer prevention as one of the key areas, Central and South-Eastern European Member States are less able to invest in this [28]. Most novel technologies, such as those in cancer care, are too expensive to be implemented in some Member States. Additionally, improving health literacy, which is seen as one of the most important investments in cancer care, is not mentioned in any of the documents we analysed [28]. Health literacy is linked to the digitalisation of care, yet another area that the Commission has plans in but misses the existing digital divide across Europe [29]. This digital divide has been amplified and further highlighted by the COVID-19 pandemic, e.g., through the use of digital certificates [30].

Persuasion can lead to common knowledge, ‘epistemic conventions’, or homogenisation of interests: the gap between actors’ understandings close when persuasion is successful [31]. The Commission’s messages were tailored to appeal to the audience of Member States, NGOs, academics, and others. It could be suggested that the backgrounds of Commissioner Kyriakides, as a cancer survivor, and President Von der Leyen, as a physician, played a role in persuading the audience. Although we cannot judge whether the persuasion efforts have been successful to the fullest extent, we can argue that with the launch of Commission initiatives before and during the COVID-19 pandemic, a step towards mitigating the NCD pandemic has been taken.

The pandemic highlighted the need for EU policy to play a more important role in crisis management and potentially in other areas as well. Ironically, it is an infectious disease that is speeding up some of the ambitious plans outlined in Europe’s Beating Cancer Plan targeted at tackling NCDs, such as giving more mandate to EU institutions for the joint procurement of medicines. It was the pandemic that led to a new and more central role for the ECDC and EMA in providing a joint European COVID-19 strategy.

## 5. Limitations

There are several limitations to this study due to the nature of content analysis. Firstly, because of the reliance on in vivo coding, a large number of unique codes were identified. This implies a high level of abstraction and simplification, which can potentially result in misclassification or missing context. However, this was mitigated by constant member checking and discussion until consensus was reached to eliminate bias. Moreover, because a lot of the analysed documents were similar to each other, it is unlikely that significant misclassifications were made. A second limitation is that only documents published on the Commission’s and European Parliament’s websites were included. This could potentially mean that relevant documents might have been missed. Finally, this study might be difficult to replicate, due to the high publication rate on behalf of the Commission and the qualitative nature of this research. Nevertheless, the large sample size does allow for sufficient data saturation and the development of themes and codes [32].

## 6. Conclusions

This study contributes to a broader understanding of how the European Commission 2019–2024 is planning to mitigate the impact of NCDs on health systems, while taking into account the COVID-19 pandemic. Although increasing health systems resilience is high on the Commission’s agenda, it seems that there are no actionable points for Member States in terms of addressing national health policy. This implies a disconnect between the Member States’ needs and the Commission’s Plan. We recommend the Commission looks towards eliminating this observed disconnect and creating actionable points in line with Member States’ health systems’ capabilities. Little attention is paid to national health systems, their functioning in the EU, and what actions the Member States themselves can take at this point, aside from partaking in this ‘ambitious plan’ by applying for EU funding. However, the pandemic emphasised weaknesses and limitations of national policy in the case of protecting health systems against cross-border health threats. It tested the Commission’s resolve in pushing towards a European framework for cooperation. The Commission’s recommendations and vision for a European Health Union can therefore lead towards further harmonisation of health policy between Member States and the EU.

## Figures and Tables

**Table 1:** Overview of themes, codes, and frequency of their occurrence.

Theme	Code	Frequency
Health plan	Elements of Europe’s Beating Cancer Plan	434
	Implementing a modern approach to cancer	413
	Reducing inequalities	83
	Cancer as public health priority	51
	Financial backing	50
COVID-19	Lessons learnt and crisis preparedness	124
	COVID-19 crisis and challenges	117
	EU Vaccines Strategy	27
	New role EU, ECDC and EMA	22
Future direction	Building a European Health Union	134
	Health in all Policies	43
	Resilience	34
	More cross-border cooperation	24
Collaboration and solidarity	Working together on all levels	295
	Ambitious plan to beat cancer	24
	Solidarity	20
	We can do more	15
Persuasion	Personalisation	64
	Provided data	51
	Shared vision and commitment	34
	Time to act now	26
	Burden from NCDs	26

## Data Availability

Not applicable.

## Appendix A

**Table A1:** Overview of included documents.

Number	Type	Date	Title
1	Speech	15 October 2020	Keynote speech by Commissioner Kyriakides to the “Transforming Breast Cancer Together” event—“Bridging the gap in breast cancer care”
2	Speech	13 October 2020	Keynote address by Commissioner Kyriakides at the launch of the MEP Lung Health Group
3	Press release	15 December 2020	Commission welcomes political agreement on EU4Health
4	Press release	4 February 2020	European Commission launches EU-wide public consultation on Europe’s Beating Cancer Plan
5	Newsletter	20 January 2020	Newsletter 248—The EU Health Programme
6	Newsletter	30 January 2020	Newsletter 249—Cancer
7	Newsletter	10 March 2020	Newsletter 251—2020 EU Health Award
8	Newsletter	27 May 2020	Newsletter 252—EU4Health Programme
9	Newsletter	15 September 2020	Newsletter 257—Cardiovascular Health
10	Newsletter	4 November 2020	Newsletter 260—The European Health Union
11	Statement	29 May 2020	Statement by Commissioner Kyriakides ahead of World No Tobacco Day
12	Statement	9 October 2020	Statement ahead of World Mental Health Day on 10 October by Stella Kyriakides, Commissioner for Health and Food Safety
13	Statement	12 November 2020	Statement by Commissioner Kyriakides to the plenary of the European Parliament on the EU4Health Programme
14	News item	29 January 2021	Bold steps towards a stronger European Health Union for the future
15	News item	15 February 2021	Europe’s Beating Cancer Plan reflects Commission’s unfailing commitment
16	Speech	2 October 2020	European Health Forum Gastein—“Unlocking the potential of data in light of early lessons from COVID-19”
17	Speech	10 December 2019	Keynote speech by Commissioner Kyriakides at the conference on “Better Access to Cancer Treatment”
18	Speech	14 September 2020	United action for better health in Europe: 70th WHO Regional Committee for Europe—keynote speech
19	Speech	26 October 2020	Keynote speech by Commissioner Kyriakides at the EU Health Summit 2020: Time for Action
20	Speech	3 February 2021	Keynote Speech by Commissioner Kyriakides for the EPP Group Online Conference—“United Against Cancer”
21	Speech	26 October 2020	Keynote speech by Commissioner Kyriakides on “Achieving Health for All through Digital Collaboration” to the World Health Summit 2020
22	Speech	26 November 2020	Keynote Speech by Commissioner Kyriakides to the Conference “Towards Data-Driven Health: Sharing is Caring”
23	Speech	24 November 2020	Keynote Speech by Commissioner Kyriakides to The Economist’s 16th Cyprus Virtual Summit—“Europe: Putting Solidarity to the Test”
24	Speech	10 June 2020	Keynote Speech delivered by Commissioner Kyriakides at the virtual Delphi Economic Forum
25	Speech	14 October 2020	Keynote speech to the 16th World Congress on public health
26	Speech	11 November 2020	Remarks by Commissioner Stella Kyriakides at the press conference on Building a European Health Union
27	Speech	3 February 2021	Remarks by Commissioner Stella Kyriakides at the press conference on Europe’s Beating Cancer Plan
28	Speech	3 February 2021	Remarks by Vice-President Schinas at the press conference on Europe’s Beating Cancer Plan
29	Speech	20 May 2020	Remarks delivered by Commissioner Kyriakides at the Press Conference on the adoption of the Biodiversity Strategy and the Farm to Fork Strategy
30	Speech	4 February 2020	Speech by Commissioner Kyriakides—Europe’s Beating Cancer Plan: Making the Personal Political
31	Speech	4 February 2020	Speech by President von der Leyen at the Europe’s Beating Cancer Plan conference
32	Speech	16 September 2020	State of the Union Address by President von der Leyen at the European Parliament Plenary
33	Press release	5 February 2021	SAMIRA Action Plan: Radiological and nuclear technology in support of Europe’s Beating Cancer Plan
34	Press release	18 December 2019	Italy: the EIB supports MolMed investment in research, development and manufacturing for cancer and rare diseases innovative treatments
35	Press release	3 February 2021	Europe’s Beating Cancer Plan: A new EU approach to prevention, treatment and care
36	Press release	15 October 2020	EU backs Swedish cancer treatment research with quasi equity investment
37	Press release	22 September 2020	Beating cancer: Better protection of workers against cancer-causing chemicals
38	Policy document	1 December 2019	Mission Letter to Commissioner Stella Kyriakides
39	Report	September 2020	Proposed Mission—Conquering Cancer: Mission Possible -Report of the Mission Board for Cancer
40	Policy document	3 February 2021	Commission staff working document stakeholder consultation—synopsis report accompanying the Communication from the Commission to the European Parliament and the Council Europe’s Beating Cancer plan
41	Policy document	3 February 2021	Communication from the Commission to the European Parliament and the Council Europe’s Beating Cancer Plan
42	Policy document	3 February 2021	Annexes to the Communication from the Commission to the European Parliament and the Council Europe’s Beating Cancer plan
43	Policy document	28 May 2020	Annexes to the Proposal for a Regulation of the European Parliament and of the Council on the establishment of a Programme for the Union’s action in the field of health—for the period 2021–2027 and repealing Regulation (EU) No 282/2014 (“EU4Health Programme”)
44	Policy document	28 May 2020	Proposal for a Regulation of the European Parliament and of the Council on the establishment of a Programme for the Union’s action in the field of health—for the period 2021–2027 and repealing Regulation (EU) No 282/2014 (“EU4Health Programme”)
